# Amplification, Inference, and the Manifestation of Objective Classical Information

**DOI:** 10.3390/e24060781

**Published:** 2022-06-01

**Authors:** Michael Zwolak

**Affiliations:** Biophysical and Biomedical Measurement Group, Microsystems and Nanotechnology Division, Physical Measurement Laboratory, National Institute of Standards and Technology, Gaithersburg, MD 20899, USA; mpz@nist.gov

**Keywords:** quantum-to-classical transition, quantum Darwinism, decoherence, amplification, inference, Holevo, quantum Chernoff bound

## Abstract

Our everyday reality is characterized by objective information—information that is selected and amplified by the environment that interacts with quantum systems. Many observers can accurately infer that information indirectly by making measurements on fragments of the environment. The correlations between the system, S, and a fragment, F, of the environment, E, is often quantified by the quantum mutual information, or the Holevo quantity, which bounds the classical information about S transmittable by a quantum channel F. The latter is a quantum mutual information but of a classical-quantum state where measurement has selected outcomes on S. The measurement generically reflects the influence of the remaining environment, E/F, but can also reflect hypothetical questions to deduce the structure of SF correlations. Recently, Touil et al. examined a different Holevo quantity, one from a quantum-classical state (a quantum S to a measured F). As shown here, this quantity upper bounds any accessible classical information about S in F and can yield a tighter bound than the typical Holevo quantity. When good decoherence is present—when the remaining environment, E/F, has effectively measured the pointer states of S—this accessibility bound is the accessible information. For the specific model of Touil et al., the accessible information is related to the error probability for optimal detection and, thus, has the same behavior as the quantum Chernoff bound. The latter reflects amplification and provides a universal approach, as well as a single-shot framework, to quantify records of the missing, classical information about S.

## 1. Introduction

The emergence of objective, classical information from quantum systems is due to amplification: Many pieces of the environment—e.g., many photons—each interact with a quantum system and acquire an imprint of certain states, the pointer states. This is the process by which select information becomes redundant and accessible to many different observers. The framework, where the environment decoheres systems and acts as a communication channel for the resulting information, is known as quantum Darwinism [1,2,3,4,5,6,7,8,9,10,11,12,13,14,15,16,17,18,19,20]. It is the pointer states that survive the interaction with the environment and create “copies” of themselves from which observers can infer the pointer state of the system. This process has been seen experimentally in both natural [21] and engineered [22,23] settings, and both theory and practical calculations are steadily progressing [24,25,26,27,28,29,30,31,32,33,34,35,36,37,38].

Within this framework, one primary question concerns the information available within an environment fragment as its size increases. This allows one to quantify redundancy: If small fragments F of the environment E all contain the same information about the system S, then that information is available to many observers. Given a global state, $\rho_{SE}$, the accessible information

(1) $$I_{\mathrm{acc}}\left(\Pi_{S}\right)=\max_{\Pi_{F}}I\left(\Pi_{S}:\Pi_{F}\right)$$

can quantify the amount of information an observer learns about $\Pi_{S}$ (a positive operator-valued measure, a POVM, on S) by making a measurement $\Pi_{F}$ on only F. The quantity $I\left(\Pi_{S}:\Pi_{F}\right)$ is the classical mutual information computed from the joint probability distribution from outcomes of $\Pi_{S}$ and $\Pi_{F}$. The POVM $\Pi_{S}$ has elements $\pi_{s}$ that generate an ensemble $\left\{\left(p_{s},\rho_{F\left|s\right.}\right)\right\}$ of outcomes *s* with probability $p_{s}={\mathrm{tr}}_{SE}\pi_{s}\rho_{SE}$ and conditional states $\rho_{F\left|s\right.}={\mathrm{tr}}_{SE/F}\pi_{s}\rho_{SE}/p_{s}={\mathrm{tr}}_{SE/F}\sqrt{\pi_{s}}\rho_{SE}\sqrt{\pi_{s}}/p_{s}$ on F (i.e., assuming the POVM acts on only S and an auxiliary system but F is not directly affected). Allowing $\Pi_{S}$ to be arbitrary, the accessible information, Equation (1), depicts a situation where some auxiliary system A, perhaps a special observer or another part of the environment, has access directly only to S, makes a measurement $\Pi_{S}$, and holds a record of the outcome *s*, leaving a joint state (after tracing out the now irrelevant S)

(2) $$\sum_{s}p_{s}{|s\rangle }_{A}\langle s|⊗\rho_{F\left|s\right.}.$$

An observer O then wants to predict the outcome *s* by making measurements only on F, e.g., correlations are generated between A and O but indirectly from separate measurements on S and F, for which Equation (1) quantifies this capability. One could then maximize the accessible information over all $\Pi_{S}$ to see what quantity the observer can learn most about. This allows one to quantify the structure of correlations between S and F induced by, e.g., a decohering interaction between them.

Within the context of physical processes that give rise to quantum Darwinism, $\Pi_{S}$ is not arbitrary, however. For redundant information to be present, there must be at least two records of some information, which, when decoherence is the main interaction, will be the pointer information. Hence, there must be an F that almost, to a degree we want to quantify, makes a measurement of the pointer states. At the same time, the remaining part of the environment, E/F, has already made an effective measurement for all intents and purposes, to a degree that we can retroactively validate. This entails that the correlations are effectively of the form of Equation (2) but with A=E/F or S and $\Pi_{S}={\hat{\Pi }}_{S}$ (the pointer observable),

(3) $$\sum_{\hat{s}}p_{\hat{s}}|\hat{s}\rangle \langle \hat{s}|⊗\rho_{F\left|\hat{s}\right.},$$

where $\hat{s}$ labels the pointer states (see Refs. [39,40] for a discussion of pointer states). This form is a consequence of “branching” [3] and appears in the good decoherence limit of purely decohering models, which will be extensively discussed below. Here, it is sufficient to note that the state, Equation (3), is the most relevant to quantum Darwinism. It makes little difference if one treats the A as E/F or as just the fully decohered, or directly measured, S, even when F is extremely large in absolute terms. Only for “global” questions, where F is some sizable fraction of the environment, does it matter. Since the environment is huge for most problems of everyday interest, such as photon scattering, F can be very large—even asymptotically large—without concern for this. However, Equation (3) does drop exponentially small corrections in the size of E/F and one can not formally take the asymptotic limit of F without first doing so in E. The degree to which asymptotic approximations work thus relies on the balance sheet—how well records are kept in the environment components compared to E’s absolute size. Ref. [14] has dealt with retaining corrections to Equation (3). Hereon, I treat the auxiliary system A as if it were S.

## 2. Results

With states of the form in Equation (3), the mutual information between A=S and F is the Holevo quantity

(4) $$\chi \left({\hat{\Pi }}_{S}:F\right)=H\left(\sum_{\hat{s}}p_{\hat{s}}\rho_{F\left|\hat{s}\right.}\right)-\sum_{\hat{s}}p_{\hat{s}}H\left(\rho_{F\left|\hat{s}\right.}\right)\equiv H_{F}-\sum_{\hat{s}}p_{\hat{s}}H_{F\left|\hat{s}\right.},$$

where $H\left(\rho \right)=-\mathrm{tr}\rho \log_{2}\rho$ is the von Neumann entropy for the state ρ. This quantity upper bounds the capacity of F to transmit pointer state information (the variable $\hat{s}$ is encoded in the conditional states $\rho_{F\left|\hat{s}\right.}$). Moreover, for an important class of interactions—purely decohering Hamiltonians with independent environment components—the quantum Chernoff bound determines the behavior of the optimal measurement on F to extract ${\hat{\Pi }}_{S}$ and, thus, is related to the accessible information, Equation (1) with $\Pi_{S}={\hat{\Pi }}_{S}$. One can generalize Equation (4) by allowing one to maximize over measurements on the system,

(5) $$\chi (\overset{ˇ}{S}:F)=\max_{\Pi_{S}}\chi \left(\Pi_{S}:F\right),$$

where, when good decoherence has taken place, $\Pi_{S}={\hat{\Pi }}_{S}$ maximizes the Holevo quantity [14]. The good decoherence limit is when E/F is sufficient to decohere the system and, thus, the SF state is exactly of the form in Equation (3) [10,14]. Here, I employ the notation $\overset{ˇ}{A}$ of Touil et al. [38] to indicate that the Holevo quantity is maximized over measurements on A, see also the next equation.

Touil et al. [38] examined an alternative Holevo quantity with the measurement on the fragment side,

(6) $$\chi (S:\overset{ˇ}{F})=\max_{\Pi_{F}}\chi \left(S:\Pi_{F}\right)=\max_{\Pi_{F}}\left[H_{S}-\sum_{f}p_{f}H_{\left.S\right|f}\right],$$

where the maximization is over all POVMs $\Pi_{F}$ and *f* labels the outcomes of $\Pi_{F}$ and $p_{f}$ their probabilities. In that work, they compute the quantum mutual information, the Holevo quantity in Equation (4), and the alternative Holevo quantity in Equation (6) for a “c-maybe” model of decoherence of S by E, a model that falls into the class of purely decohering models (see below). They analytically found $\chi (S:\overset{ˇ}{F})$ by making use of the Koashi–Winter monogamy relation [41] and showed all the mutual information quantities above that approach the missing information, $H_{S}$, with a similar dependence on F.

If one were to interpret this alternative Holevo quantity, Equation (6), in the typical way, then it would bound the channel capacity of S to transmit information about (the optimal) $\Pi_{F}$. One important observation, however, is that, in the good decoherence limit—when the SF state is of the form in Equation (3)—$\chi \left(S:\Pi_{F}\right)$ lower bounds $\chi ({\hat{\Pi }}_{S}:F)$ for any $\Pi_{F}$ by the data processing inequality since ${\hat{\Pi }}_{S}$ is already measured on S by E/F. In this limit, $\chi (S:\overset{ˇ}{F})$ is the actual accessible pointer information.

For an arbitrary SF state, however, there is no strict relation of $\chi \left(\Pi_{S}:F\right)$ or $\chi (\overset{ˇ}{S}:F)$ with $\chi \left(S:\Pi_{F}\right)$ or $\chi (S:\overset{ˇ}{F})$. In that case, the Holevo quantities with measurements on the F side can not upper or lower bound quantities with S side measurements. For a particular state with a given inequality between F and S side measurements, one can swap S and F in the state $\rho_{SF}$—it is arbitrary after all—and reverse the inequality. Instead, the inequality

(7) $$\chi (S:\overset{ˇ}{F})\geq I_{\mathrm{acc}}\left(\Pi_{S}\right)$$

holds for any $\Pi_{S}$. The measurement on the two sides of the inequality is generically different—the measurement that maximizes $\chi (S:\overset{ˇ}{F})$ is not the measurement, $\Pi_{F}^{☆}$, that maximizes $I\left(\Pi_{S}:\Pi_{F}\right)$ to get the accessible information, Equation (1). The proof of Equation (7) is straightforward,

$$\begin{array}{cc} \chi (S:\overset{ˇ}{F}) & =\max_{\Pi_{F}}\chi \left(S:\Pi_{F}\right)\\ \; & \geq \chi (S:\Pi_{F}^{☆})\\ \; & =\chi (MS:\Pi_{F}^{☆})\\ \; & \geq \chi (\Pi_{S}:\Pi_{F}^{☆})\\ \; & =I_{\mathrm{acc}}\left(\Pi_{S}\right), \end{array}$$

where the system M is adjoined in a product state with $\rho_{SF}$ and a unitary on MS makes a measurement $\Pi_{S}$. The fourth line follows from data processing.

Equation (7) is an accessibility bound. Any information about S (i.e., that can be extracted by a direct POVM on S) can, at best, have $\chi (S:\overset{ˇ}{F})$ amount of shared information with F. Then, as already noted, if the good decoherence limit is reached, that bound becomes equality,

(8) $$\overset{\mathrm{Good}\;\mathrm{Decoherence}}{\chi (S:\overset{ˇ}{F})=I_{\mathrm{acc}}({\hat{\Pi }}_{S}),}$$

for the pointer information. This follows from the form of the state in Equation (3). To determine $\chi (S:\overset{ˇ}{F})$ for this state, an apparatus makes a measurement $\Pi_{F}$ and records the outcome, leaving a joint system-apparatus state $\sum_{\hat{s},f}p_{\hat{s}}|\hat{s}\rangle \langle \hat{s}|⊗p_{\left.f\right|\hat{s}}\left|f\rangle \langle f\right|$. This is a classical-classical state that yields, after maximizing over $\Pi_{F}$, both $\chi (S:\overset{ˇ}{F})$, Equation (6), and the accessible information, Equation (1). This makes $\chi (S:\overset{ˇ}{F})$ desirable in the context of quantum Darwinism: It not only is a better bound on the accessible information in the good decoherence limit—the main limit of interest for quantum Darwinism—but it is the actual accessible information.

To proceed further—to compute the accessible information and the associated redundancy—we need to specify a model or class of models that provide the global states of interest. The everyday photon environment has a particular structure where independent environment components (photons) scatter off objects, acquire an imprint of the state, and transmit that information onward, interacting little with each other in the process [11,12,16,42,43,44]. This structure is captured by purely decohering Hamiltonians by independent environment components. I will consider this general class here. Under this evolution, the quantum Chernoff bound (QCB) provides a universal lower bound to the accessible information and the associated redundancy. The quantum Chernoff result is also meaningful on its own as a single-shot result, quantifying how well an individual observer (with the best measurement apparatus) can learn the pointer state of S indirectly from F.

Pure decoherence occurs when environments select, but do not perturb, the pointer states of S. When the environment components do so independently, the Hamiltonian is of the form

(9) $$H=H_{S}+{\hat{\Pi }}_{S}\sum_{k=1}^{{}^{♯}\;E}\Upsilon_{k}+\sum_{k=1}^{{}^{♯}\;E}\Omega_{k}$$

with $[{\hat{\Pi }}_{S},H_{S}]=0$ and the initial state

(10) $$\rho \left(0\right)=\rho_{S}\left(0\right)⊗\left[\overset{{}^{♯}\;E}{\underset{k=1}{⨂}}\rho_{k}\left(0\right)\right].$$

Here, *k* specifies a component of the environment E of size ${}^{♯}\;E$. The operators, $\Upsilon_{k}$ and $\Omega_{k}$, are arbitrary. This class of models contains the c-maybe model of Touil et al. [38]. That model has ${\hat{\Pi }}_{S}=0\cdot \left|0\rangle \langle 0\right|+1\cdot \left|1\rangle \langle 1\right|$ and $\exp\left[ı\Upsilon_{k}t\right]=\sin a|0\rangle \langle 0|+\cos a\left(|0\rangle \langle 1|+|1\rangle \langle 0|\right)-\sin a|1\rangle \langle 1|$ for all *k*, where *a* is the angle of rotation of the “target” environment bit after a time *t*. Note that all the coupling frequencies (i.e., the energy scales divided by the reduced Planck’s constant) are absorbed into the definition of the operators $H_{S}$, $\Upsilon_{k}$, and $\Omega_{k}$, while ${\hat{\Pi }}_{S}$ is dimensionless. All other operators are 0. The collection of operators act similarly to those in the controlled NOT gate. They only swap as well, only a bit more lazily, as here *a* is any number, so it is called c-maybe.

Starting from the initial product state, Equation (10), and evolving for some time under the Hamiltonian, Equation (9), one can obtain the conditional states that appear in the Holevo quantity, Equation (4),

(11) $$\rho_{F\left|\hat{s}\right.}=\underset{k\in F}{⨂}\rho_{k\left|\hat{s}\right.}.$$

Due to the structure of the evolution, these are product states over the components of the environment fragment. However, they need not be identically distributed (that is, they need not be fully i.i.d.—independently and identically distributed—states).

The structure, Equation (11), is a manifestation of amplification. The pointer states $\hat{s}$ leave an imprint on the environment components, of which there are many. Observers intercepting those environment components can then make a measurement to infer the pointer state. This is the setting of quantum hypothesis testing. For instance, in the binary case with two pointer states $\hat{s}=0$ or 1, one wants to decide whether the fragment state is $\rho_{\left.F\right|0}$ or $\rho_{\left.F\right|1}$ with a minimum average probability of error, $P_{e}=p_{\hat{s}=0}\mathrm{tr}\Pi_{\left.F\right|1}\rho_{\left.F\right|0}+p_{\hat{s}=1}\mathrm{tr}\Pi_{\left.F\right|0}\rho_{\left.F\right|1}$. This is based on a POVM measurement, $\Pi_{F}$, composed of two positive operators $\Pi_{\left.F\right|0}$ and $\Pi_{\left.F\right|1}$ (with $\Pi_{\left.F\right|0}+\Pi_{\left.F\right|1}=I$) that indicate the occurrence of “0” or “1”, respectively. The first contribution to this average error is when the actual state is $\rho_{\left.F\right|0}$, with *a priori* probability of occurring $p_{\hat{s}=0}$ (where I explicitly show $\hat{s}=0$ to connect to Equation (3)) but the measurement yielded the incorrect outcome $\Pi_{\left.F\right|1}$. Similarly for the second contribution. Moreover, when amplification occurs, i.e., the conditional states are of the form in Equation (11), one is specifically interested in how the error probability behaves as the fragment size grows. This is the setting of the QCB.

To employ the QCB, one makes use of a two-sided measurement. The first is on S, putting it into its pointer states (i.e., $\chi ({\hat{\Pi }}_{S}:F)$ now provides the mutual information between S and F). This reflects the action of E/F and is the good decoherence limit—, i.e., ${}^{♯}\;E\to \infty$ provided S and E have interacted for some finite time under the evolution given by Equations (9) and (10). This also requires that the coupling strength to the environment components do not depend on ${}^{♯}\;E$. The second is on F to access the pointer state. By Fano’s inequality [45,46],

(12) $$\chi ({\hat{\Pi }}_{S}:F)\geq I_{\mathrm{acc}}({\hat{\Pi }}_{S})\geq H_{S}-h\left(P_{e}\right)-P_{e}\ln\left[D-1\right],$$

where $P_{e}$ is the error probability for extracting information about a (sub)space of pointer states (of dimension *D*) from a measurement on F. One could replace the left hand side of this inequality with $\chi (\overset{ˇ}{S}:F)\geq \chi ({\hat{\Pi }}_{S}:F)$. Here, I use the binary entropy, $h\left(x\right)=-x\log_{2}x-\left(1-x\right)\log_{2}\left(1-x\right)$. The QCB upper bound, $P_{e}^{☆}\geq P_{e}$, gives a second inequality

(13) $$I_{\mathrm{acc}}({\hat{\Pi }}_{S})\geq H_{S}-h\left(P_{e}\right)-P_{e}\ln\left[D-1\right]\geq H_{S}-h\left(P_{e}^{☆}\right)-P_{e}^{☆}\ln\left[D-1\right],$$

which is partway to the final QCB result [16,19].

The QCB upper bounds the error probability, $P_{e}^{☆}\geq P_{e}$, for both the D=2 case [47,48,49] or the D>2 cases [50]. There is no fundamental difference between these cases, it is only the closest two states that determine the asymptotic decay of $P_{e}$ when D>2. I will restrict to D=2 from hereon to make a correspondence with Touil et al. [38]. The error probability (bound) is

(14) $$P_{e}^{☆}=\min_{0\leq c\leq 1}p_{1}^{c}p_{2}^{1-c}\prod_{k\in F}\mathrm{tr}\left[\rho_{\left.k\right|1}^{c}\rho_{\left.k\right|2}^{1-c}\right].$$

For pure SE states in the purely decohering scenario, Equations (9) and (10), *c* can be any value between 0 and 1 within the generalized overlap contribution, $\mathrm{tr}\left[\rho_{\left.k\right|1}^{c}\rho_{\left.k\right|2}^{1-c}\right]$, and it will give the exact overlap ${\left|\langle \psi_{\left.k\right|1}|\psi_{\left.k\right|2}\rangle \right|}^{2}={\left|\gamma_{k}\right|}^{2}$ (which is also the decoherence factor $\gamma_{k}$ squared for this case of pure states). Touil et al. [38] consider the homogeneous case where $\gamma_{k}=\gamma$ for all *k*, which I will also consider (see Refs. [16,19] for inhomogeneous results).

For pure states, therefore, only the prefactor needs optimizing over *c* as the generalized overlap gives ${\left|\gamma \right|}^{2{}^{♯}\;F}$ for all 0≤c≤1 and with ${}^{♯}\;F$ the number of components in F. The prefactor is optimal at one of the two boundaries (c=0 or c=1), giving

(15) $$P_{e}^{☆}=\min\left[p_{1},p_{2}\right]{\left|\gamma \right|}^{2{}^{♯}\;F}.$$

I use a slightly different notation here than Ref. [38] to keep the correspondence with prior work. Opposed to pure states, for mixed SE states within the pure decohering scenario, Equations (9) and (10), the error probability (bound) is $\sqrt{p_{1}p_{2}}\prod_{k\in F}\mathrm{tr}\left[\rho_{\left.k\right|1}^{1/2}\rho_{\left.k\right|2}^{1/2}\right]$ for both spin and photon models [16,19] (i.e., c=1/2 is optimal). Either prefactor, $\min\left[p_{1},p_{2}\right]$ or $\sqrt{p_{1}p_{2}}$, will give a bound for the pure state case. Letting the prefactor to be just some *C*, the QCB result for pure, homogeneous SE is

(16) $$I_{\mathrm{acc}}({\hat{\Pi }}_{S})\geq H_{S}-h\left(C{\left|\gamma \right|}^{2{}^{♯}\;F}\right)\equiv X_{QCB},$$

where I stress that this is a classical-classical information about random variable $\hat{s}$ (pointer states on S) with measurement outcomes on F. If we want general SE states, but still the pure decoherence model, Equations (9) and (10), we have exactly the same form as Equation (16) but the decoherence factor (the pure state overlap) is replaced by the generalized measure of overlap, $\mathrm{tr}\left[\rho_{\left.k\right|1}^{1/2}\rho_{\left.k\right|2}^{1/2}\right]$, see Ref. [19] for these expressions in terms of generic angles (between conditional states) and lengths on the Bloch sphere for spins and Ref. [16] for photons.

The QCB is a universal result. The bound Equation (14) is true for all models of pure decoherence by independent spins or the standard photon model, all dimensions in between (qutrits, qudits, etc.), inhomogeneous models, pure and mixed SE states, and ones with individual self-Hamiltonians on E. The only stipulation for Equation (14) and the lower bound $H_{S}-H(P_{e}^{☆})$ is that one is distinguishing within a two-dimensional subspace of S pointer states. For higher dimensional subspaces, the number of pointer states, *D*, appears in Equation (13) and the exponent in the decay of $P_{e}^{☆}$ requires a pair-wise minimization of the generalized overlap over conditional states (as well as a different prefactor outside of the exponential).

The most important aspect of the compact form, Equation (16), and its generalization to higher *D*, is that the right hand side reflects actual, inferable information about the pointer states that the observer can retrieve by interaction with just F *in a single shot*. Moreover, while the QCB is traditionally cast as an asymptotic result, we have not actually used any asymptotic limits to obtain Equation (16). Both of these aspects—single shot and finite F—provide a natural setting for our world, where observers are “agents” within these regimes. One can then ask questions about resources of observers (for instance, global versus local measurements on F subcomponents [51] or the ability to perform coherent measurements [52]) that further refine the results but do not change the fundamental framework of single-shot, finite F inference.

Let us return to the c-maybe model and the Holevo quantities. Touil et al. [38] present results for the quantum mutual information, $\chi (\overset{ˇ}{S}:F)$, and $\chi (S:\overset{ˇ}{F})$. In the good decoherence limit, the latter two are

(17) $$\begin{array}{ccc} \chi (\overset{ˇ}{S}:F) & = & -\frac{1}{2}\log_{2}\left[p_{1}p_{2}\left(1-{\left|\gamma \right|}^{2{}^{♯}\;F}\right)\right]\\ & & -\sqrt{1-4p_{1}p_{2}\left(1-{\left|\gamma \right|}^{2{}^{♯}\;F}\right)}{\mathrm{Arctanh}}_{2}\left[\sqrt{1-4p_{1}p_{2}\left(1-{\left|\gamma \right|}^{2{}^{♯}\;F}\right)}\right] \end{array}$$

and

(18) $$\chi (S:\overset{ˇ}{F})=H_{S}+\frac{1}{2}\log_{2}\left[p_{1}p_{2}{\left|\gamma \right|}^{2{}^{♯}\;F}\right]+\sqrt{1-4p_{1}p_{2}{\left|\gamma \right|}^{2{}^{♯}\;F}}{\mathrm{Arctanh}}_{2}\left[\sqrt{1-4p_{1}p_{2}{\left|\gamma \right|}^{2{}^{♯}\;F}}\right]$$

in the form as they appear in their main text but using the notation here (Equations (17) and (20) in Ref. [38]). Rewriting these in terms of binary entropy gives

(19) $$\chi (\overset{ˇ}{S}:F)=h\left[\frac{1}{2}\left(1+\sqrt{1-4p_{1}p_{2}\left(1-{\left|\gamma \right|}^{2{}^{♯}\;F}\right)}\right)\right],$$

corresponding to the good decoherence expressions in Ref. [10], and

(20) $$\chi (S:\overset{ˇ}{F})=H_{S}-h\left[\frac{1}{2}\left(1+\sqrt{1-4p_{1}p_{2}{\left|\gamma \right|}^{2{}^{♯}\;F}}\right)\right].$$

We see that Equations (16) and (20) have a similar structure. Indeed, in the good decoherence limit and for pure conditional states, the accessible information, which is equivalent to Equation (18) or Equation (20), is equal to $H_{S}-h\left(P_{e}\right)$. Here, $P_{e}=\frac{1}{2}\left(1-\mathrm{tr}\left|p_{1}\rho_{\left.F\right|1}-p_{2}\rho_{\left.F\right|2}\right|\right)$ is the optimal error probability, which is given by the Helstrom measurement [53], for single shot state discrimination of the conditional fragment states [54,55,56]. This is not true for mixed or for higher dimensional pointer subspaces [57,58,59,60]. It can be verified in this case by a direct computation of the error probability from the optimal measurement for the pure conditional states. For $\rho_{F\left|\hat{s}\right.}$ pure, the trace distance in the Helstrom expression just requires diagonalizing an operator in a two-dimensional subspace, giving

(21) $$P_{e}=\frac{1}{2}\left(1-\sqrt{1-4p_{1}p_{2}{\left|\gamma \right|}^{2{}^{♯}\;F}}\right)$$

(this readily generalizes to the inhomogeneous case: The factor ${\left|\gamma \right|}^{2{}^{♯}\;F}$ just needs to be replaced by $\prod_{k\in F}{\left|\gamma_{k}\right|}^{2}$). This result makes no use of the fact that the environment components were spins, and thus it is directly applicable to (pure state) photon scattering off an object in a two dimensional superposition, more directly supporting the connection discussed in Touil et al. [38] and extending it to $\chi (S:\overset{ˇ}{F})$ in the good decoherence limit. Moreover, as with the QCB result, the form of the accessible information for pure SE states, $H_{S}-h\left(P_{e}\right)$, with the optimal $P_{e}$ from Equation (21) holds regardless of the environment components. They can be spins, qudits, or photons. Furthermore, the connection with hypothesis testing allows for even more general statements about models that are not purely decohering. For instance, for projection-valued measurements and pure SE states, one obtains the same accessible information, $H_{S}-h\left(P_{e}\right)$, but the error probability just has the overlap between the conditional fragment states, which can behave in a manner that is not exponentially decaying with ${}^{♯}\;F$.

While specific to the case of D=2 and pure SE states evolving under Equations (9) and (10), the connection provides a window into the behavior of different ways to quantify correlations. The alternate Holevo quantity, $\chi (S:\overset{ˇ}{F})$, becomes the inferable information in this specific setting. However, inferable information has a universal form that goes beyond this specific setting of dimensionality and purity.

*Redundancy*. The decay to the classical plateau—the missing information $H_{S}$ about the system—for the quantities in Equations (16), (19) and (20), all are controlled by the F-induced decoherence factor, $\gamma^{2{}^{♯}\;F}$. Ultimately, though, we are interested in the redundancy of information. This requires introducing a control, the information deficit δ, which reflects the fact that one can not generally obtain perfect knowledge from a finite-size fragment F. This is typically taken as

(22) $$X\left(F\right)\geq H_{S}\left(1-\delta \right),$$

where $X\left(F\right)$ is some mutual information (quantum mutual information, Holevo, accessible information, etc.). This is the form I will employ here. However, both the form of the QCB and the form of $\chi (S:\overset{ˇ}{F})$ (in the good decoherence limit) suggest employing the information deficit as an entropic quantity when thresholding entropic measures of information,

(23) $$X\left(F\right)\geq H_{S}-H\left[\delta \right].$$

This allows δ to be a factor reflecting distinguishability of conditional states and allows for non-asymptotic computations to proceed for the redundancy (it removes the transcendental form of the equations). I will not use this in what follows.

The approach to the plateau and the redundancy (to within δ) have simple asymptotic results regardless of quantity used to compute them. The decay exponent to the plateau, ξ, of some information theoretic quantity $X\left(F\right)$, such as Equations (19) and (20), or Equation (16), is

(24) $$\xi =-\lim_{{}^{♯}\;F\to \infty }\frac{1}{{}^{♯}\;F}\ln\left[H_{S}-X\left(F\right)\right].$$

For the pure, homogeneous c-maybe model, all three decay to the plateau with exponent

(25) $$\xi =-\ln{\left|\gamma \right|}^{2}.$$

*That is universality in a nutshell*. Moreover, the exponent is the leading order of the redundancy,

(26) $$R_{\delta }≃{}^{♯}\;E\frac{\xi }{\ln1/\delta }={}^{♯}\;E\frac{\ln{\left|\gamma \right|}^{2}}{\ln\delta }.$$

This is the essence of the QCB: The exponent—the quantum Chernoff information, $\xi_{QCB}$, or its inhomogeneous counterpart, ${\bar{\xi }}_{QCB}$—controls the redundancy, see Refs. [16,19] for additional discussion and results. For the pure c-maybe model, this exponent is the same whether using Equations (19) and (20), or Equation (16). The quantum mutual information also yields the same decay and redundancy in the good decoherence limit, as it is the same as $\chi (\overset{ˇ}{S}:F)$ from Equation (19). In order to apply Equation (24) for the quantum mutual information, one needs ${}^{♯}\;E\to \infty$. As already mentioned previously, though, this will entail good decoherence provided some finite interaction between S and E components has taken place. In the end, all the information theoretic quantities provide the same decay and redundancy, which the asymptotic calculation, Equation (24), makes apparent in a non-empirical manner.

**Figure 1:**
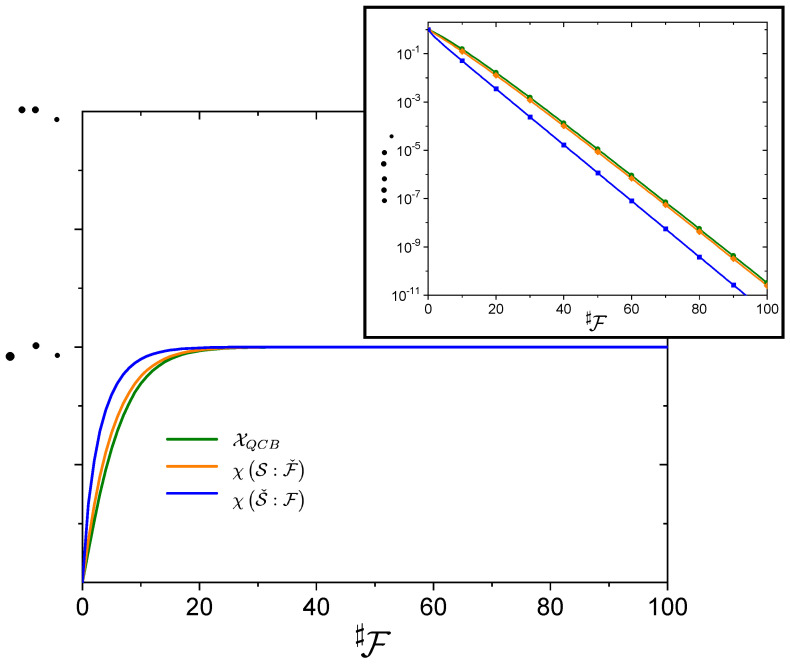
**Approach to the plateau.** Information measures X versus fragment size ${}^{♯}\;F$ for $p_{1}=1/4$ and γ=7/8. All three quantities, $X=X_{QCB}$ (green line), $\chi (S:\overset{ˇ}{F})$ (orange line), and $\chi (\overset{ˇ}{S}:F)$ (blue line), rapidly rise to the classical plateau, $H_{S}$, as the fragment size ${}^{♯}\;F$ increases. The quantum mutual information, $I\left(S:F\right)$ (not shown), is equivalent to $\chi (\overset{ˇ}{S}:F)$ when good decoherence is present. The QCB result, $X_{QCB}$, lower bounds the other two, but is close to $\chi (S:\overset{ˇ}{F})$. The inset shows the decay to the plateau. All three measures decay with the same exponent. The $\chi (\overset{ˇ}{S}:F)$ does, though, deviate from the other two quantities, as the latter two have a prefactor that depends on ${}^{♯}\;F$ (both with the same functional form). This offset does not influence the redundancy asymptotically (i.e., as a relative correction, it itself decays).

Figure 1 shows the approach to the plateau for the three information measures. The quantity $\chi (\overset{ˇ}{S}:F)$ is a weaker bound to the accessible information. Yet, the separation between the decay curves is unimportant for passing the threshold in Equation (22): $\chi (\overset{ˇ}{S}:F)$ passes it sooner than the other quantities, but this only gives a relative correction to Equation (26) that goes to zero asymptotically (${}^{♯}\;F$ and −lnδ have to simultaneously go to infinity), albeit weakly as 1/lnδ. To clarify this statement, let $R_{\delta }=R_{\delta }^{\circ }+R_{\delta }^{\prime }$, with $R_{\delta }^{\circ }$ from the right hand side of Equation (26) and $R_{\delta }^{\prime }$ the corrections. The relative correction, $R_{\delta }^{\prime }/R_{\delta }^{\circ }$ decays as 1/lnδ for $\chi (\overset{ˇ}{S}:F)$ and as $\ln\left(\ln1/\delta \right)/\ln\delta$ for $\chi (S:\overset{ˇ}{F})$ and $X_{QCB}$ as δ→∞. In other words, $R_{\delta }^{\prime }∼1/{\left(\ln\delta \right)}^{2}$ asymptotically. The very weak prefactor, $\ln\left(\ln1/\delta \right)$, for the latter two cases is due to the presence of ${}^{♯}\;F$ in the prefactor in Equations (28) and (29). The leading order contribution to the decay for $\chi (\overset{ˇ}{S}:F)$ is

(27) $$\frac{p_{1}p_{2}\log_{2}\frac{p_{2}}{p_{1}}}{p_{2}-p_{1}}{\left|\gamma \right|}^{2{}^{♯}\;F}$$

or with a prefactor of 1/2ln2 when $p_{1}=p_{2}=1/2$. For $\chi (S:\overset{ˇ}{F})$, the decay is

(28) $$p_{1}p_{2}\log_{2}\left[\frac{e}{p_{1}p_{2}}{\left|\gamma \right|}^{-2{}^{♯}\;F}\right]{\left|\gamma \right|}^{2{}^{♯}\;F}$$

and, for the QCB result,

(29) $$C\log_{2}\left[\frac{e}{C}{\left|\gamma \right|}^{-2{}^{♯}\;F}\right]{\left|\gamma \right|}^{2{}^{♯}\;F}$$

with $C=\min\left[p_{1},p_{2}\right]$ or $\sqrt{p_{1}p_{2}}$ depending on whether we take the pure state result or generically take the mixed state bound. These forms show the same exponential decay but the latter two have a weak dependence of the prefactor on ${}^{♯}\;F$.

## 3. Conclusions

Quantum Darwinism clarifies the role of the proliferation of information in the quantum-to-classical transition. Here, I examined the quantity introduced by Touil et al. [38], $\chi (S:\overset{ˇ}{F})$, where an (optimal) measurement is made on the fragment, reminiscent of the quantum Chernoff bound. It provides an appealing approach to finding the redundancy of information, as it is an accessibility bound that becomes the accessible information in the limit of good decoherence. For the special case of a pure SE state, the accessible information is directly related to the optimal error probability for distinguishing conditional states on the environment (i.e., hypothesis testing or inference), of which an exact expression (including the prefactor) can be computed. Moreover, this connection immediately generalizes the result to any pure, D=2 model (spin environments, qudit environments, photon environments, etc.) and to inhomogeneous environments (including ones with self-Hamiltonians, as in Equation (9)). That decay, as expected, has the same exponent as the QCB, as the QCB promises (and only promises) to yield the right asymptotic decay, not the prefactor. Asymptotic analysis provides a non-empirical way to show that all quantities give the same redundancy—due to the same exponent—to leading order (and that corrections are small) and makes the universality of the plateau approach manifest. Since the QCB applies more generally, its universal bound should further help shed light on future results that yield exact entropic quantities or alternative bounds. Its importance—the QCB’s importance—goes beyond this, however, as it provides a single shot, finite F framework for understanding how we observers learn in a quantum Universe.

## Data Availability

Not applicable.

## References

[1] Ollivier H., Poulin D., Zurek W.H. (2004). Objective Properties from Subjective Quantum States: Environment as a Witness. Phys. Rev. Lett..

[2] Ollivier H., Poulin D., Zurek W.H. (2005). Environment as a witness: Selective proliferation of information and emergence of objectivity in a quantum universe. Phys. Rev. A.

[3] Blume-Kohout R., Zurek W.H. (2005). A Simple Example of “Quantum Darwinism”: Redundant Information Storage in Many-Spin Environments. Found. Phys..

[4] Blume-Kohout R., Zurek W.H. (2006). Quantum Darwinism: Entanglement, branches, and the emergent classicality of redundantly stored quantum information. Phys. Rev. A.

[5] Zurek W.H. (2007). Relative States and the Environment: Einselection, Envariance, Quantum Darwinism, and the Existential Interpretation. arXiv.

[6] Blume-Kohout R., Zurek W.H. (2008). Quantum Darwinism in Quantum Brownian Motion. Phys. Rev. Lett..

[7] Zurek W.H. (2009). Quantum Darwinism. Nat. Phys..

[8] Zwolak M., Quan H.T., Zurek W.H. (2009). Quantum Darwinism in a Mixed Environment. Phys. Rev. Lett..

[9] Paz J.P., Roncaglia A.J. (2009). Redundancy of classical and quantum correlations during decoherence. Phys. Rev. A.

[10] Zwolak M., Quan H.T., Zurek W.H. (2010). Redundant imprinting of information in nonideal environments: Objective reality via a noisy channel. Phys. Rev. A.

[11] Riedel C.J., Zurek W.H. (2010). Quantum Darwinism in an Everyday Environment: Huge Redundancy in Scattered Photons. Phys. Rev. Lett..

[12] Riedel C.J., Zurek W.H. (2011). Redundant information from thermal illumination: Quantum Darwinism in scattered photons. New J. Phys..

[13] Riedel C.J., Zurek W.H., Zwolak M. (2012). The rise and fall of redundancy in decoherence and quantum Darwinism. New J. Phys..

[14] Zwolak M., Zurek W.H. (2013). Complementarity of quantum discord and classically accessible information. Sci. Rep..

[15] Zurek W.H. (2014). Quantum Darwinism, classical reality, and the randomness of quantum jumps. Phys. Today.

[16] Zwolak M., Riedel C.J., Zurek W.H. (2014). Amplification, Redundancy, and Quantum Chernoff Information. Phys. Rev. Lett..

[17] Korbicz J.K., Horodecki P., Horodecki R. (2014). Objectivity in a Noisy Photonic Environment through Quantum State Information Broadcasting. Phys. Rev. Lett..

[18] Brandão F.G.S.L., Piani M., Horodecki P. (2015). Generic emergence of classical features in quantum Darwinism. Nat. Commun..

[19] Zwolak M., Riedel C.J., Zurek W.H. (2016). Amplification, Decoherence and the Acquisition of Information by Spin Environments. Sci. Rep..

[20] Zwolak M., Zurek W.H. (2017). Redundancy of einselected information in quantum Darwinism: The irrelevance of irrelevant environment bits. Phys. Rev. A.

[21] Unden T.K., Louzon D., Zwolak M., Zurek W.H., Jelezko F. (2019). Revealing the Emergence of Classicality Using Nitrogen-Vacancy Centers. Phys. Rev. Lett..

[22] Ciampini M.A., Pinna G., Mataloni P., Paternostro M. (2018). Experimental signature of quantum Darwinism in photonic cluster states. Phys. Rev. A.

[23] Chen M.C., Zhong H.S., Li Y., Wu D., Wang X.L., Li L., Pan J.W. (2019). Emergence of classical objectivity of quantum Darwinism in a photonic quantum simulator. Sci. Bull..

[24] Balanesković N. (2015). Random unitary evolution model of quantum Darwinism with pure decoherence. Eur. Phys. J. D.

[25] Horodecki R., Korbicz J.K., Horodecki P. (2015). Quantum origins of objectivity. Phys. Rev. A.

[26] Giorgi G.L., Galve F., Zambrini R. (2015). Quantum Darwinism and non-Markovian dissipative dynamics from quantum phases of the spin-1/2 XX model. Phys. Rev. A.

[27] Balanesković N., Mendler M. (2016). Dissipation, dephasing and quantum Darwinism in qubit systems with random unitary interactions. Eur. Phys. J. D.

[28] Knott P.A., Tufarelli T., Piani M., Adesso G. (2018). Generic Emergence of Objectivity of Observables in Infinite Dimensions. Phys. Rev. Lett..

[29] Milazzo N., Lorenzo S., Paternostro M., Palma G.M. (2019). Role of information backflow in the emergence of quantum Darwinism. Phys. Rev. A.

[30] Campbell S., Çakmak B., Müstecaplıoğlu O., Paternostro M., Vacchini B. (2019). Collisional unfolding of quantum Darwinism. Phys. Rev. A.

[31] Roszak K., Korbicz J.K. (2019). Entanglement and objectivity in pure dephasing models. Phys. Rev. A.

[32] García-Pérez G., Chisholm D.A., Rossi M.A.C., Palma G.M., Maniscalco S. (2020). Decoherence without entanglement and quantum Darwinism. Phys. Rev. Res..

[33] Lorenzo S., Paternostro M., Palma G.M. (2020). Anti-Zeno-based dynamical control of the unfolding of quantum Darwinism. Phys. Rev. Res..

[34] Ryan E., Paternostro M., Campbell S. (2021). Quantum Darwinism in a structured spin environment. Phys. Lett. A.

[35] Kiciński M., Korbicz J.K. (2021). Decoherence and objectivity in higher spin environments. Phys. Rev. A.

[36] Korbicz J.K. (2021). Roads to objectivity: Quantum Darwinism, Spectrum Broadcast Structures, and Strong quantum Darwinism—A review. Quantum.

[37] Kwiatkowski D., Cywiński L., Korbicz J.K. (2021). Appearance of objectivity for NV centers interacting with dynamically polarized nuclear environment. New J. Phys..

[38] Touil A., Yan B., Girolami D., Deffner S., Zurek W.H. (2022). Eavesdropping on the Decohering Environment: Quantum Darwinism, Amplification, and the Origin of Objective Classical Reality. Phys. Rev. Lett..

[39] Zurek W.H. (1981). Pointer basis of quantum apparatus: Into what mixture does the wave packet collapse?. Phys. Rev. D.

[40] Zurek W.H. (1982). Environment-induced superselection rules. Phys. Rev. D.

[41] Koashi M., Winter A. (2004). Monogamy of quantum entanglement and other correlations. Phys. Rev. A.

[42] Joos E., Zeh H.D. (1985). The emergence of classical properties through interaction with the environment. Z. Phys. B Condens. Matter.

[43] Joos E., Zeh H.D., Kiefer C., Giulini D.J., Kupsch J., Stamatescu I.O. (2003). Decoherence and the Appearance of a Classical World in Quantum Theory.

[44] Schlosshauer M.A. (2007). Decoherence and the Quantum-to-Classical Transition.

[45] Cover T.M., Thomas J.A. (2006). Elements of Information Theory.

[46] Nielsen M.A., Chuang I.L. (2011). Quantum Computation and Quantum Information.

[47] Audenaert K.M.R., Calsamiglia J., Munoz-Tapia C.R., Bagan E., Masanes L., Acin A., Verstraete F. (2007). Discriminating States: The Quantum Chernoff Bound. Phys. Rev. Lett..

[48] Audenaert K.M.R., Nussbaum M., Szkoła A., Verstraete F. (2008). Asymptotic Error Rates in Quantum Hypothesis Testing. Commun. Math. Phys..

[49] Nussbaum M., Szkoła A. (2009). The Chernoff lower bound for symmetric quantum hypothesis testing. Ann. Stat..

[50] Li K. (2016). Discriminating quantum states: The multiple Chernoff distance. Ann. Stat..

[51] Kincaid J., Zwolak M. Phases of observation and the universal accessibility of classical reality.

[52] Blume-Kohout R., Croke S., Zwolak M. (2013). Quantum data gathering. Sci. Rep..

[53] Helstrom C.W. (1976). Quantum Detection and Estimation Theory.

[54] Levitin L.B., Belavkin V.P., Hirota O., Hudson R.L. (1995). Optimal Quantum Measurements for Two Pure and Mixed States. Quantum Communications and Measurement.

[55] Fuchs C.A. (1995). Distinguishability and Accessible Information in Quantum Theory. Ph.D. Dissertation.

[56] Ban M., Osaki M., Hirota O. (1996). Upper bound of the accessible information and lower bound of the Bayes cost in quantum signal-detection processes. Phys. Rev. A.

[57] Sasaki M., Barnett S.M., Jozsa R., Osaki M., Hirota O. (1999). Accessible information and optimal strategies for real symmetrical quantum sources. Phys. Rev. A.

[58] Shor P.W. (2000). On the Number of Elements Needed in a POVM Attaining the Accessible Information. arXiv.

[59] Shor P.W. (2003). The Adaptive Classical Capacity of a Quantum Channel, or Information Capacities of Three Symmetric Pure States in Three Dimensions. arXiv.

[60] Barnett S.M., Croke S. (2009). Quantum state discrimination. Adv. Opt..

